# Intranasal treatment of lixisenatide attenuated emotional and olfactory symptoms via CREB-mediated adult neurogenesis in mouse depression model

**DOI:** 10.18632/aging.202358

**Published:** 2021-01-10

**Authors:** Guoyong Ren, Pan Xue, Bin Wu, Fei Yang, Xuemei Wu

**Affiliations:** 1 Department of Neurology, General Hospital of TISCO, Sixth Hospital of Shanxi Medical University, Taiyuan, China

**Keywords:** lixisenatide, depression, olfactory function, neurogenesis, CREB

## Abstract

Convergent lines of evidence indicate a striking correlation between olfactory deficits and depressive symptoms. However, the effectiveness of intranasal treatment of antidepressant or other neurotrophic agents remains poorly understanding. Here in this study, we created depression mouse model and explored the antidepressant effects of GLP-1 analog lixisenatide (LXT) with intranasal treatment. Consecutive intranasal treatment of LXT remarkably reduced the depressive and anxiety behaviors. Meanwhile, it also improved the olfactory memory and olfactory sensitivity. Immunofluorescent analysis demonstrated the LXT improved the adult neurogenesis in olfactory system and hippocampus. Inhibition of adult neurogenesis with TMZ caused the compromised effects of LXT in improving emotional and olfactory functions, suggesting the vital role of adult neurogenesis in LXT induced depression therapeutic effects. Treatment of LXT resulted in the increased phosphorylation of CREB protein in hippocampal tissue, indicating CREB plays important roles in antidepressant effects of LXT intranasal treatment. Inhibiting CREB with chemical approach decreased effects of LXT in reserving depression induced emotional and olfactory functions. In conclusion, our study suggests intranasal treatment of LXT could be a potential antidepressant to improve the olfactory functions as well as the emotional behaviors.

## INTRODUCTION

In recent years, depression became the main psychiatric disorder to cause the disabilities. Patients suffer not only the depressive and anxiety mood, but also display the combined symptoms e.g. olfactory deficits, sleep disorders and even short-term memory loss [1]. Olfactory dysfunctions are major symptoms combined with depression. Clinical reports indicate a striking olfactory dysfunction in correlating with emotional symptoms in depression patients [2]. Animal study demonstrated that olfactory bulbectomy could be utilized to mimic depression symptoms [3]. On the other hand, corticosterone induced depression mice model displayed the impaired olfactory functions [4]. Thus, improving olfactory functions is also recognized as the main task in antidepressant therapy. It was reported that antidepressants like fluoxetine and other natural compounds could attenuate the emotional abnormalities and olfactory dysfunctions simultaneously [5, 6]. However, it was not clear whether targeting on improving olfactory functions could benefit the antidepressant process.

Adult neurogenesis is the key neural plasticity to regulates emotional and olfactory functions [7]. Neural stem cells (NSCs) resident at subventricular zone (SVZ) and hippocampus remain its proliferative activity and continuously differentiate into functional neurons, which are recognized to regulate the olfactory functions and antidepressant behaviors. NSCs in SVZ develop into neural progenitors and migrate to olfactory bulb to participate the encoding of olfactory memories. As in hippocampus, NSCs get commitment into adult neurons and regulates emotional functions by interacting with local circuit network. Thus, improving adult neurogenesis is the key target for antidepressant treatment and olfactory functional recovery as well. Intranasal treatment of the antidepressant might also effective in promoting neurogensis in olfactory system as thereby gain the antidepressant effects. It was widely reported that analogs of incretin glucagon-like peptide-1 (GLP-1) cannot only serve as the metabolic regulators but also play as the important neuroprotective agent. lixisenatide (LXT) a.k.a Adlyxin is the GLP-1 analog anti-diabetic medication. It mimics the effects of GLP-1 to control the blood glucose level [8]. It was reported that LXT could improvement behavioral functions in neurodegenerative diseases like Alzheimer’s disease and Parkinson’s disease [9, 10]. However, as one of the medication in anti-diabetic treatments, peripheral treatment of LXT or other metabolic drugs might adversely affect the blood glucose level. Other adverse clinical effects such as nausea, vomiting, and diarrhea were also reported [8]. Herein, a new treatment pathway is necessary for further apply LXT into neurological disorders. Given the importance of olfactory functions as well as the olfactory neurogenesis in depression treatment. Whether intranasal administration of LXT could obtain the antidepressant functions remains unknown. Exploring its effects and underlying mechanism could provide a new therapeutic method with better safety in antidepressant clinical practice.

In this study, we created depression mouse model and tested the effects of intranasal treatment of LXT in improving olfactory and emotional symptoms. The adult neurogenesis in olfactory system and hippocampus were also detected. We further identified that signaling protein cAMP response element binding protein (CREB) regulated adult neurogenesis mediates the antidepressant effects of LXT intransal treatment.

## RESULTS

### Intranasal treatment of LXT reserved CUMS induced emotional and olfactory dysfunctions

**Figure 1:**
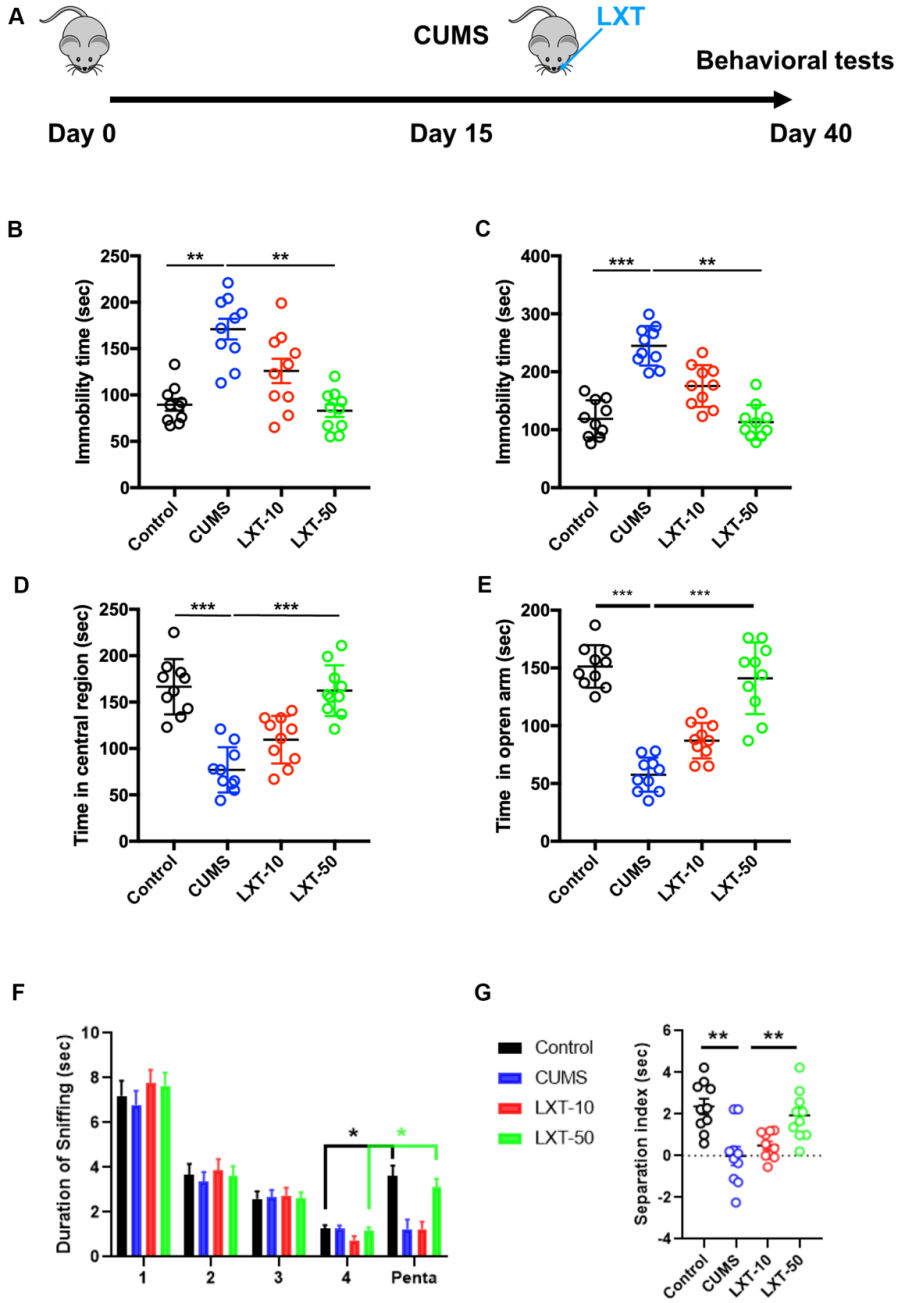
**Intranasal treatment of LXT reserved CUMS induced emotional and olfactory dysfunctions.** (**A**) Schematic picture to show the experimental process. Mice were performed with CUMS protocol and conducted with the intranasal administration of the LXT. (**B**, **C**) Immobility time of the mice to show the antidepressant behaviors in FST and TST. (**D**) Time of the mice to spend in center area of the region in open field test. (**E**) Time of mouse to spend in open arm in elevated plus maze test. (**F**, **G**) Duration of the sniff and the separation index (sniff duration of pentanol) of mice in olfactory discrimination test.

We first created CUMS model for mimicking depression symptoms. LXT with two dosages (10 nmol/kg/d and 50 nmol/kg/d) were administrated by intranasal treatment from day-15 to day-40 (Figure 1A). Depression and anxiety behaviors were then detected. FST and TST demonstrated that 50 nmol/kg/d treatment of LXT significantly reduced the immobility time, indicating the effects of LXT intranasal treatment to depressive symptoms. Anxiety behaviors were assessed with open field test (OFT) and elevated plus maze (Figure 1B, 1C, one-way ANOVA, p<0.01 LXT-50 vs. CUMS). In open field test, treatment of LXT intranasally resulted the remarkable prolonged movement in center area (Figure 1D, one-way ANOVA, p<0.001 LXT-50 vs. CUMS). Consistently, LXT treatment also increased the movement duration of the mice in open arm area in EPM (Figure 1F, one-way ANOVA, p<0.001 LXT-50 vs. CUMS). Olfactory dysfunctions also related with the emotional symptoms in depression [6]. Herein, we tested the olfactory discrimination behavior to evaluate the olfactory functions. Mice were repeatedly exposed to butanol (Buta) and the sniffing duration was gradually decreased. As expected, sniffing duration was dramatically prolonged when the mice was exposed to pentanol (Penta), indicating the olfactory discrimination ability. The CUMS model showed dramatic decreased of the separation index (sniffing duration of Pento/sniffing duration of Pento and last Buto*100%) in compared with control (Figure 1H, one-way ANOVA, p<0.01, CUMS vs. Control). While administration of LXT by intranasal treatment recovered the olfactory function, which reflected by the significant increased separate index to CUMS (Figure 1H, one-way ANOVA, p<0.01, CUMS vs. LXT-50). Therefore, LXT intranasal treatment could improve the emotional and olfactory functions in depression animal model.

### LXT intranasal treatment enhanced adult neurogenesis in hippocampus and olfactory bulbs

**Figure 2:**
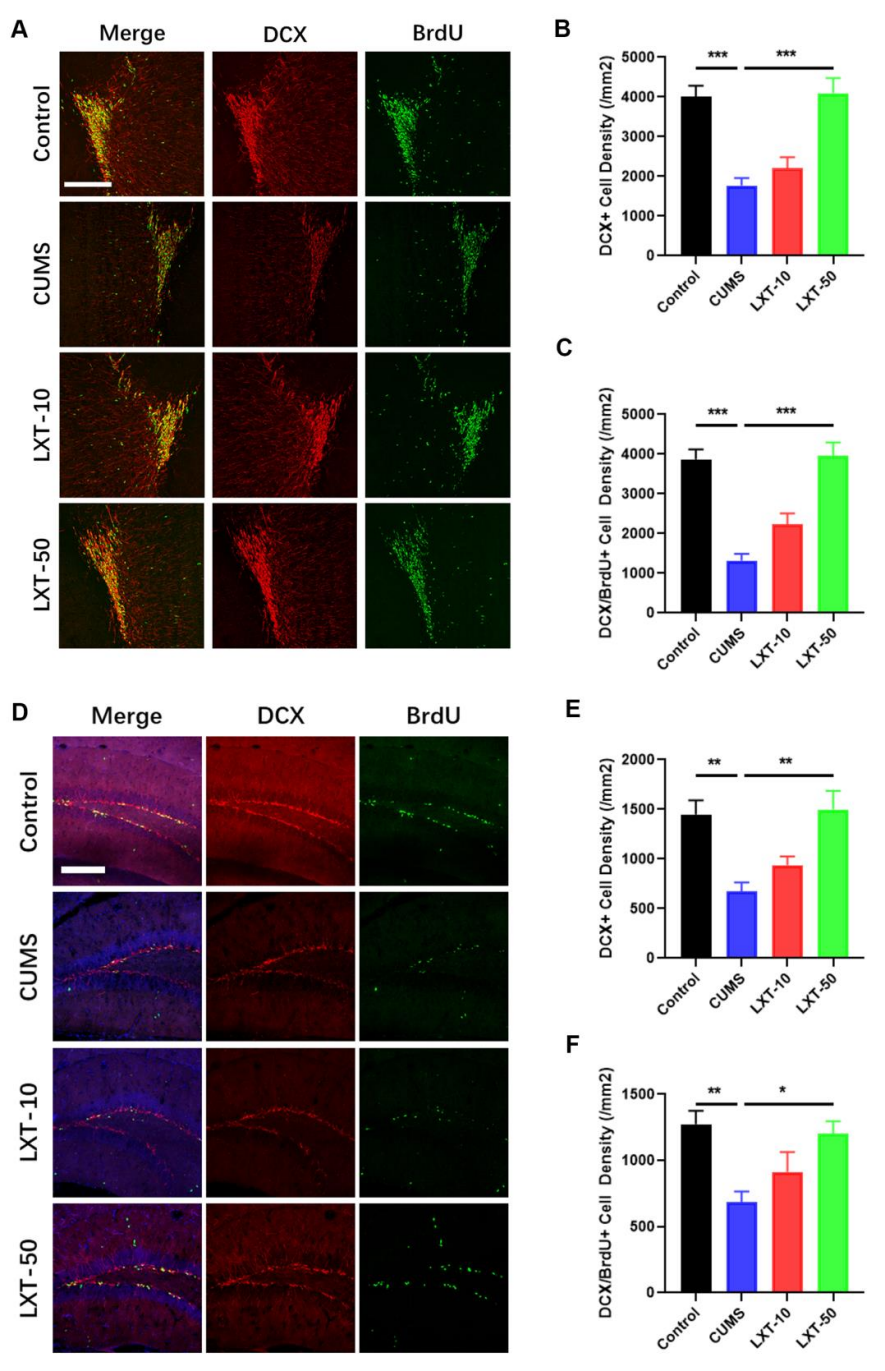
**LXT intranasal treatment enhanced adult neurogenesis in hippocampus and olfactory bulbs.** (**A**, **D**) Confocal image to show the distribution and cell number of DCX+ immature neurons (Red) and BrdU+ (Green) newborn cells in olfactory bulb and hippocampal DG region. (**B**, **C**) DCX+ immature neuron cell density and the newborn DCX+/BrdU+ cell density in olfactory bulb was recovered by LXT treatment. (**E**, **F**) DCX+ immature neuron cell density and the newborn DCX+/BrdU+ cell density in hippocampal DG region was recovered by LXT treatment.

Adult neurogenesis at olfactory bulbs (OB) and hippocampus plays the critical roles in regulating olfactory functions and emotional behaviors [4]. To further explore whether adult neurogenesis is correlated with the effects of LXT to improve the behavioral dysfunctions in CUMS model, we labelled the new generated cells with BrdU and co-stained the BrdU with immature neuronal marker doublecortin (DCX) at both OB and hippocampal DG region. As the result shown, BrdU+ cells as well as BrdU and DCX dual positive cells were considerably decreased at OB region in CUMS mice (Figure 2A–2C, one-way ANVOA, p<-0.001 CUMS vs. Control). LXT treatment increased the number of both BrdU+ cells and BrdU+/DCX+ cells (Figure 2A–2C, one-way ANVOA, p<-0.001 CUMS vs. LXT-50). The same condition was observed in hippocampal region. Intranasal treatment of LXT with 50mg/kg/d resulted in the significant increased BrdU+ cells and BrdU+/DCX+ cells in hippocampus, when comparing with CUMS model (Figure 2E, 2F, one-way ANOVA, p<0.001 CUMS vs. LXT-50). The results indicate that LXT treatment could improve the adult neurogenesis in both OB and hippocampal region. Such biological effects might involve with the behavioral improvement of LXT to CUMS animal model.

### Hippocampal neurogenesis is required in effects of LXT to CUMS model

**Figure 3:**
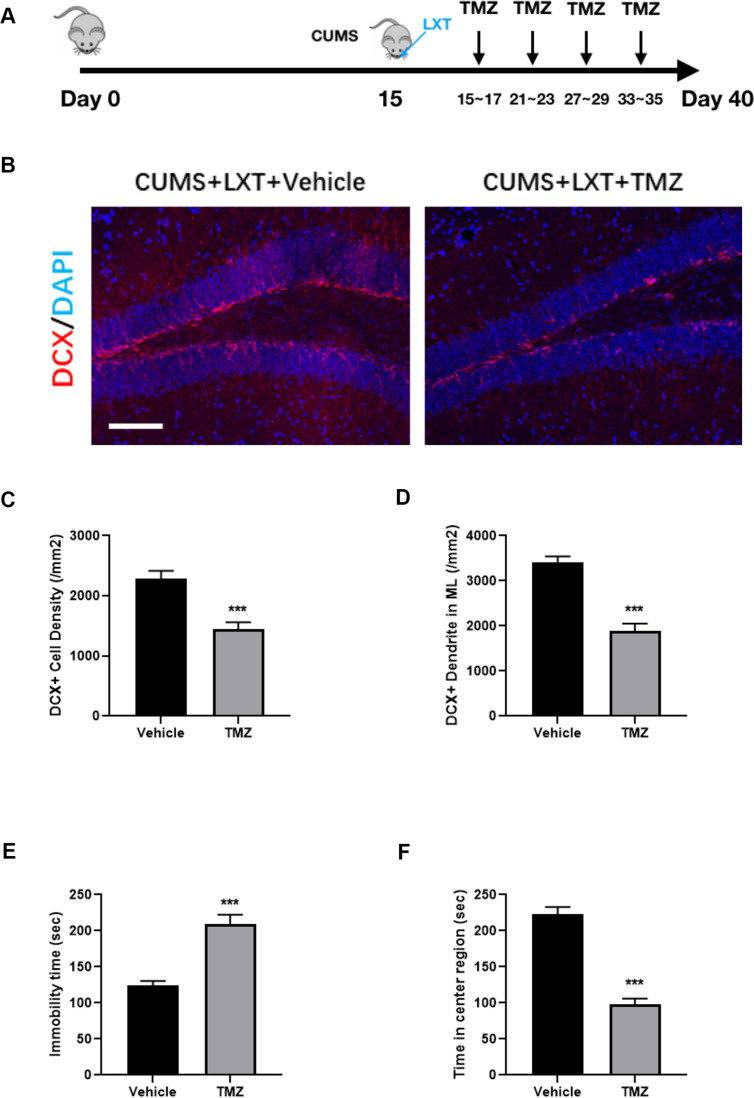
**Hippocampal neurogenesis is required in effects of LXT to CUMS model.** (**A**) Schematic picture to show the experimental process. Mice with CUMS protocol was conducted with TMZ treatment and combined with the intranasal administration of the LXT. (**B**) Confocal image to show the distribution and cell number of DCX+ immature neurons (Red). (**C**, **D**) Statistical analysis of DCX+ immature neurons and DCX+ neural fibers. (**E**, **F**) Statistical analysis of immobility time in FST as well as the time spend in center region in open field test.

The antimitotic drug temozolomide (TMZ) has previously been used successfully as an experimental tool in animals to deplete adult neurogenesis and is used regularly as a standard chemotherapy for brain cancer [11]. Here we treated the mice with TMZ to inhibit the adult neurogenesis (Figure 3A). Treatment of TMZ remarkably decreased the adult neurogenesis. It was shown that density of DCX+ cells in hippocampus was dampened (Figure 3B, 3C, student t-test, p<0.001). Moreover, the dendritic fibers labelled with DCX were also dramatically decreased after TMZ treatment (Figure 3B, 3D, student t-test, p<0.001). Those results indicate that TMZ exerted the inhibitory effects to adult neurogenesis in hippocampus. As the consequence, inhibition of hippocampal neurogenesis compromised the behavioral improvement of the LXT. Injection of TMZ increased the immobility time in FST and simultaneously decreased the movement duration in center region in OFT (Figure 3E, 3F, student t-test, p<0.001). Therefore, LXT intranasal treatment ameliorated the depression and anxiety like behaviors depending on the enhancement of neurogenesis.

### CREB mediates the effects of LXT to improve adult neurogenesis and related behaviors

**Figure 4:**
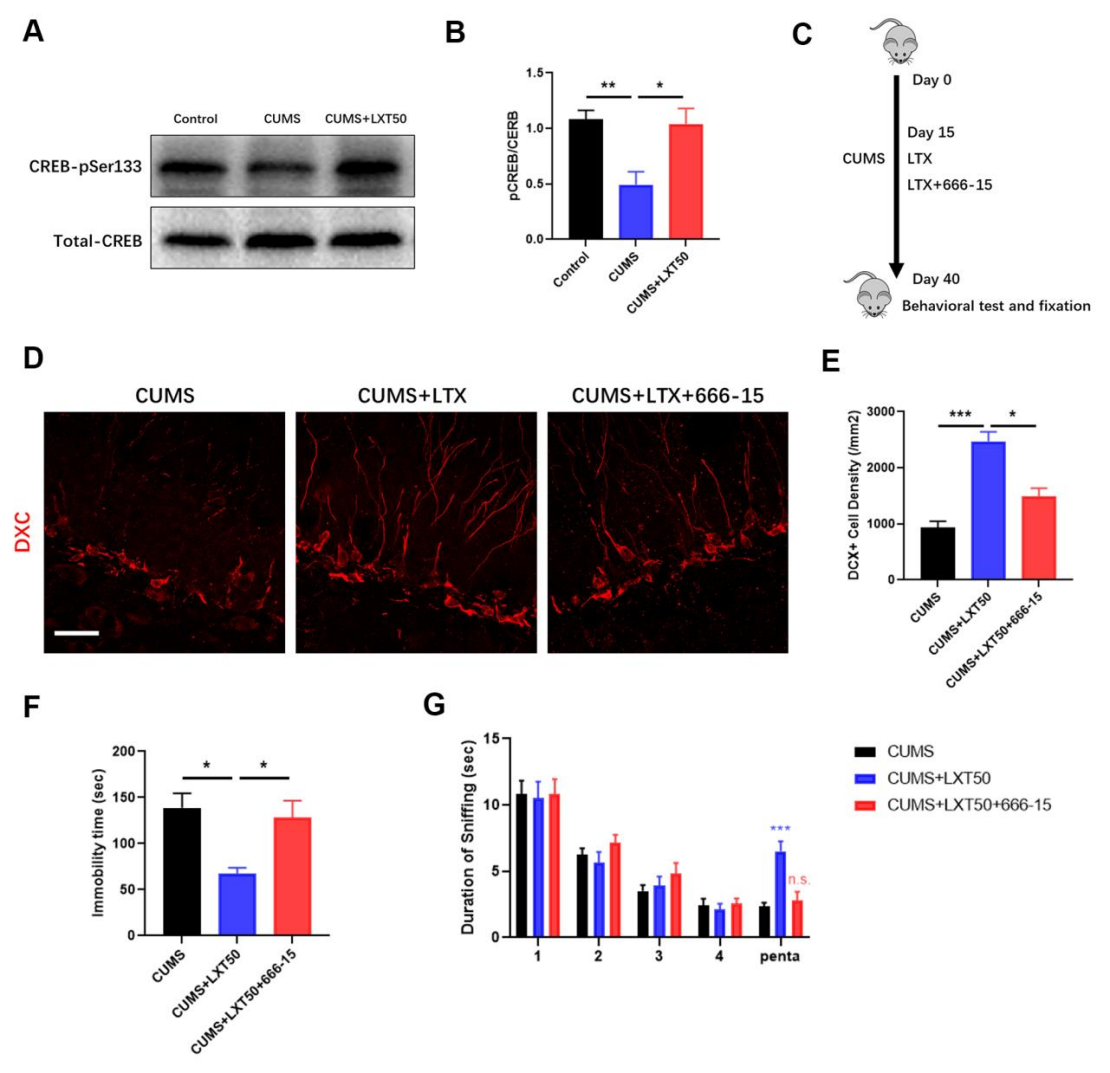
**CREB mediates the effects of LXT to improve adult neurogenesis and related behaviors.** (**A**, **B**) Western blot band and statistical analysis to show the phosphorylation of CREB at pSer333 and the total protein level. (**C**) Schematic picture to show the experimental process. Mice with CUMS protocol was conducted with 666-15 treatment and combined with the intranasal administration of the LXT. (**D**) Confocal image to show the distribution and cell number of DCX+ immature neurons (Red) in hippocampal DG region. (**E**) Statistical analysis of DCX+ immature neuronal density. (**F**) Statistical analysis of immobility time in FST. (**G**) Statistical analysis of the duration of the sniff and the separation index (sniff duration of pentanol) of mice in olfactory discrimination test.

CREB pathway is the critical signaling to regulate adult neurogenesis. In hippocampal tissue, we observed that LXT intranasal treatment significantly elevated the CREB phosphorylation level at Ser133 in comparing with CUMS mode, which suggesting LXT recovered the activity of CREB (Figure 4A, 4B, one-way ANOVA, p<0.05, LXT50 vs. CUMS). This result indicates that CREB might be one of the important regulators involving with the effects of LXT to improve behaviors via promoting neurogenesis. To further confirm the role of CREB signaling in therapeutic effects of LXT intranasal treatment, we injected the treatment mice with 666-15 to inhibit the activation of CREB (Figure 4C, 4E). By labelling the immature neurons in hippocampal DG region with DCX, we detected that 666-15 administration to LXT treatment group remarkably decreased the density of DCX+ immature neuronal density (Figure 4D, 4E, one-way ANOVA, p<0.05 CUMS+LXT50 vs. CUSM+LXT50+666-15). Behavioral test showed that 666-15 treatment prolonged the immobility time in FST and inhibited the duration of sniffing to Penta in olfactory discrimination test (Figure 4F, one-way ANOVA, p<0.05 CUMS+LXT50 vs. CUSM+LXT50+666-15; G, two-way ANVOA, p<0.001, CUMS+TLX50 trail 4 vs. Penta; in significance, p=0.9937, CUMS+TLX50+666-15 trail 4 vs. Penta). These two behavioral tests indicate that inhibition of CREB signaling blocked the behavioral effects of LXT with intranasal treatment. Thus, CREB mediated adult neurogenesis the critical mechanism underlying the effects of LXT intranasal treatment to depression.

## DISCUSSION

If intranasal treatment has equal therapeutic effects, it could avoid the adverse effects of the antidepressants including liver and kidney toxicity, digestive symptoms and obesity. The main discovery of current study demonstrated the therapeutic effects of LXT to CUMS induced depression model. Moreover, our results also suggested the effectiveness of intranasal treatment of LXT in promoting adult neurogenesis. CREB-mediate adult neurogenesis plays the critical role in regulating the effects of LXT intranasal treatment to emotional and olfactory functions.

Previous publications indicated that GLP-1 analogs have significant neuroprotective effects in neurodegenerative diseases including Alzheimer’s disease (AD), Parkinson’s disease (PD) and stroke [12, 13]. The effectiveness of incretins to neurodegenerations as well as close relationship between neurodegenerations and metabolic disorders suggest the possibility to use anti-diabetic drugs for neuroprotection. Indeed, the intranasal treatment of insulin has been proved could attenuate the symptoms in AD and mild cognitive impairment [14]. Improvement of olfactory functions was also indicated as the alternative strategy for antidepressant therapy [15]. Treatment of antidepressant including fluoxetine and natural compound baicalin were also demonstrated could improve the olfactory and depressive like symptoms in depression model [4, 6]. In this study, we used intranasal treatment of LXT to CUMS induced depression model. Long-term intranasal treatment of LXT improved the olfactory functions and obtained the antidepressant effects (Figure 1). Oral or injection administration of GLP-1 analogs or GLP-1R agonists were demonstrated with the antidepressant and pro-neurogenesis effects in depression model [16, 17]. Our behavioral results suggest that intranasal treatment of the GLP-1 analogs may also have the behavioral protection against depression.

Olfactory dysfunction is highly associated with the development of depressive symptoms. It was reported that depression model displayed the dysfunctional neurogenesis in both hippocampus and olfactory system, which related with the emotional symptoms and olfactory deficits, respectively [18]. Thus, improving neurogenesis in olfactory system and hippocampus are equally important to attenuate the depression related symptoms. In our study, intranasal treatment of the LXT increased the neuronal generation in both OB and hippocampal DG region (Figure 2). The increased newborn neurons in OB and hippocampus match the improved olfactory functions as well as the antidepressant behaviors. Further experiment using TMZ showed that blockage of the neurogenesis compromised the effects of LXT to improve antidepressant behaviors (Figure 3). Intranasal treatment of the LXT could improve the adult neurogenesis in olfactory system and hippocampus. More importantly, improvement of the neurogenesis plays the key role in regulating LXT mediate the antidepressant behaviors. Previous studies indicate the critical roles of adult neurogenesis in emotional regulatory effects of the antidepressants. It was reported that ketamine could also improve the antidepressant behaviors in dependent with its improving effects to adult hippocampal neurogenesis [19]. Likewise, classic antidepressants like fluoxetine performs the behavioral regulation in depend on improvement of adult neurogenesis [20]. Our study further indicates the possibility to use metabolic regulators as the antidepressant through intranasal treatment.

CREB is the important signaling in regulating neural plasticity. Dysfunctional CREB mediated synaptic plasticity were considered plays the vital roles in neurological and psychiatric diseases [21, 22]. CREB can response to neurotrophic signaling and regulate the adult neurogenesis including survival, maturation and integration of new neurons [23]. With the inhibitor of CREB 666-15, we detected the prohibited neurogenesis in hippocampus as well as the decreased antidepressant effects of LXT intranasal treatment (Figure 4). The improvement effects of LXT in olfactory and emotional functions were all reduced by CREB administration. In conclusion, the CREB-mediated adult neurogenesis plays the key role in regulating the antidepressant effects of LXT. It was reported that CREB/CRTC2-dependent transcriptional pathway is critical for regulating glucose homeostasis by controlling production of GLP-1 [24]. Combined with the effects of TMZ reduced the LXT effects, our results indicate that CREB mediates the antidepressant effects of LXT by controlling adult neurogenesis (Figure 3). In conclusion, our study provides the effectiveness of intranasal treatment of LXT to reduce the depression induced emotional and olfactory dysfunctions. Moreover, CREB-mediated the adult neurogenesis plays the critical role in this effect. This study could offer the new evidence for exploring new antidepressant enabling intranasal treatment and the relative biological underpinning.

## MATERIALS AND METHODS

### Animals

C57BL/6N mice (age of 8 weeks, male) were obtained from laboratory animal center in Shanxi Medical University. Mice were maintained in the standard environment (12 h light/dark cycle, with lights on at 8:00 A.M., ad libitum access to dry food pellets and water). All animal performance were followed the guidelines of the Committee for Research and Ethical Issues of the International Association for the Study of Pain and were approved by the Animal Care and Use Committee of Shanxi Medical University Research Ethic Committee. After a 1-week adaptation period, mice were processed for the chronic unpredictable mild stress (Figure 1A). Briefly, the CUMS paradigm consisted in exposure, once daily, to one of the following aversive stressors: restraint-mice were placed in a 50 ml plastic tube (Falcon) with openings in both sides for breathing, for 1 h; shaking-groups of five mice were placed in a plastic box container and placed in an orbital shaker for 1 h at 150 rpm; social defeat-mice were introduced in a cage of an aggressive mice and after being defeated, they were placed in a transparent and perforated plastic container, to avoid further physical contact, inside the resident homecage for 30 min; hot air stream-mice were exposed to a hot air stream from a hairdryer for 10 min; overnight illumination-mice were exposed to regular room light during the night period; inverted light cycle-regular room light was off during daytime and on during nighttime for 2 days; tilted cage-homecages were tilted in a 45° angle during 1 h [25]. The process was repeated for 40 days, and the LXT treatment were conducting from the 15^th^ day of modelling process. LXT were treated based on clinical dosage and separated with dosages as 10 nmol/kg and 50 nmol/kg, dissolved in PBS and performed intranasal treatment to mice with total volume of 50μl daily. The CREB inhibitor 666-15 (10 mg/kg) was injected to the mice with i.p. every two days. Administration was conducted using a gentle pipette of 2.5 μL per nostril with no anesthetics. Temozolomide (TMZ) was treated to the mice with i.p. on a daily dosage of 20mg/kg.

### Behavioral tests

### Forced swim test

FST was conducted in a water tank (cylinder 30 cm height x 20 cm diameters). Mice were gently placed into the water and allowed for total 6 min free swimming. First 2 min was considered as the environmental habituation, and mobility time including swim and struggle in last 4 min was recorded to analyze the learned helplessness.

### Tail suspension test

Mice were suspended on the tap-top of their tails for 6 min. Distance from mice nose to the floor was 25cm. The mobility time (excluding the swim-like oscillations and pendulum) was recorded as the indicator of depressive mood.

### Open field test

OFT was processed in the 40cm×40cm×40cm white box. Mice were put into the box with allowance of free movement for 10min. The time percentage of the mice in central regions (20cm×20cm in center) was analyzed for evaluating the anxiety mood.

### Elevated plus maze

Mice were performed with EPM to further confirm the anxiety mood. Mice were placed on the platform comprises two open arms (25 x 5 x 0.5 cm) across from each other and perpendicular to two closed arms (25 x 5 x 16 cm) with a center platform (5 x 5 x 0.5 cm), allowing for move freely for 10 min. The time of the mice staying on open arm were recorded for analyzing the anxiety-mood.

### Immunofluorescence

Mice were sacrificed with cardiac perfusion by 4% paraformaldehyde (PFA). Before cardiac perfusion, mice were anaesthetized with 100-200 mg/kg body weight for Ketamine and 5-16 mg/kg body weight for Xylazine injected intraperitoneally. Brain was collected and post-fixed in 4% PFA for 24 h. Tissue was then embedded after dehydration with 30% of sucrose. Hippocampal and olfactory bulb cryosections were prepared with 20μm thickness. Briefly, sections after antigen retrieve (pH 6.0 citrate acid) and washing with 0.3% TritionX-100 PBS were incubated with 5% goat serum (0.3 TritionX-100). For BrdU staining, sections were incubated with 2M HCl for 1 h at room temperature (RT) and wash with PBST for 3 times (15min each time). Primary antibodies (Mouse-anti-BrdU, CST, 1:400; Rabbit-anti-DCX, CST, 1:400) were incubated with tissue sections at 4° C overnight. Secondary antibodies (Goat-anti-Rabbit-Alexa fluor 488, ThermoFisher, 1:800; Goat-anti-Mouse-Alexa fluor 568, ThermoFisher, 1:800) were incubated with sections for 2 h RT. DAPI (Sigma) was incubated with sections for 10min. Tissue IF images were obtained with confocal microscopy (Carl Zeiss, LSM 700) with Z-stack for 20μm. Image was shown as maximum projection of Z-stack. Required positive cell number was calculated and displayed as the cell density per mm^2^.

### Western blot

Hippocampal tissue was collected, and the protein lysate was prepared with RIPA buffer. Briefly, primary antibodies (rabbit anti-pCREB (Ser133), CST, 1:1000; rabbit anti-CREB, CST, 1:1000) were incubated with membrane at 4° C overnight. Secondary antibody (Goat-anti-rabbit HRP, CST, 1:2000) was incubated with membrane at room temperature for 1 hour. Protein bands were visualized with enhanced chemiluminescent (ECL, Pierce, Rockford, IL) and analyzed using the image system of Quantity One (Bio-Rad, Quantity One 1-D Analysis Software).

### Statistics

All values are expressed as mean ± SEM. For analyzing across multiple groups, comparisons were performed by one-way or two-way analysis of variance (ANOVA) followed by multiple comparison test via GraphPad Prism 5 (GraphPad software Inc, CA, USA). Two group comparing was performed with student t-test. A p value of 0.05 was cut off as statistically significance.
